# Important nutrient sources and carbohydrate metabolism patterns in the growth and development of spargana

**DOI:** 10.1186/s13071-024-06148-1

**Published:** 2024-02-16

**Authors:** Pei Liang, Peng Liang, Kemin Chen, Zhengxin Chen, Yamei Liu, Yujin Lin, Jin Li, Ruijia Fu, Gang Lu, Dayong Wang

**Affiliations:** 1 Laboratory of Biopharmaceuticals and Molecular Pharmacology, School of Pharmaceutical Sciences, Hainan University, Haikou, 570228 Hainan China; 2 Key Laboratory of Tropical Translational Medicine of the Ministry of Education, School of Tropical Medicine, Hainan Medicine University, Haikou, 571199 Hainan China; 3 Emerging Tropical Infectious Diseases Science Innovation Group, Hainan Medicine University, Haikou, 571199 Hainan China; 4 Department of Pathology, The Fifth People’s Hospital of Hainan Province, Haikou, 570102 Hainan China; 5 School of Biomedical Information and Engineering, Hainan Medical University, Haikou, 571199 Hainan China; 6 Department of Blood Transfusion, The People’s Hospital of Lingshui, Lingshui, 572400 Hainan China; 7 Central Laboratory, Sanya People’s Hospital, Sanya, 572000 Hainan China; 8 Key Laboratory of Tropical Biological Resources of the Ministry of Education of China, Hainan University, Haikou, 570228 Hainan China

**Keywords:** *Sparganum*, Nutrient sources, Carbohydrate metabolism, Key enzymes, Transcriptional levels, Drug targets

## Abstract

**Background:**

Sparganosis is a worldwide food-borne parasitic disease caused by spargana infection, which infects the muscle of frogs and snakes as well as many tissues and organs in humans. There are currently no viable treatments for sparganosis. Understanding spargana’s nutrition source and carbohydrate metabolism may be crucial for identifying its energy supply and establishing methods of treatment for sparganosis.

**Methods:**

Using an amino acid analyzer and nutrient concentration detection kits, we assessed nutrient concentrations in the muscles of *Fejervarya limnocharis* and *Pelophylax plancyi* infected or not infected with spargana. Quantitative polymerase chain reaction (PCR) was used to quantify the major enzymes involved in five glucose metabolism pathways of spargana developing in vivo. We also used quantitative PCR to assess key enzymes and transcriptome sequencing to explore the regulation of carbohydrate metabolic pathways in vitro in response to different 24-h food treatments.

**Results:**

Infected muscle tissues had considerably higher concentrations of glucogenic and/or ketogenic amino acids, glucose, and glycogen than non-infected muscle tissues. We discovered that the number of differentially expressed genes in Gene Ontology (GO) and Kyoto Encyclopedia of Genes and Genomes (KEGG) pathway analysis was larger in low-glucose than in other dietary groups. We examined differences in the expression of genes producing amino acid transporters, glucose transporters, and cathepsins in spargana grown in various nutritional environments. In the normal saline group, only the major enzymes in the tricarboxylic acid cycle (TCA), glycogenesis, and glycogenolysis pathways were expressed. The l-glutamine group had the greatest transcriptional levels of critical rate-limiting enzymes of gluconeogenesis and glycogenesis. Furthermore, the low-glucose group had the highest transcriptional levels of critical rate-limiting enzymes involved in the TCA, glycolytic, and glycogenolysis pathways. Surprisingly, when compared to the in vitro culturing groups, spargana developing in vivo exhibited higher expression of these critical rate-limiting enzymes in these pathways, with the exception of the pentose phosphate pathway.

**Conclusions:**

Spargana have a variety of nutritional sources, and there is a close relationship between nutrients and the carbohydrate metabolism pathways. It takes a multi-site approach to block nutrient absorption and carbohydrate metabolism pathways to provide energy to kill them.

**Graphical Abstract:**

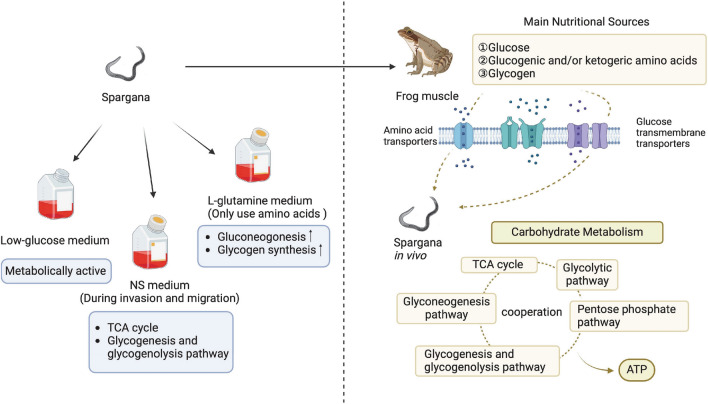

**Supplementary Information:**

The online version contains supplementary material available at 10.1186/s13071-024-06148-1.

## Background

Sparganosis is a dangerous food-borne zoonosis caused by spargana infections that poses a serious hazard to human health. Because of the diversification of the human diet, frogs, snakes, birds, and wild pigs have become common foods, and these animals are intermediate hosts for spargana, resulting in a yearly increase in the incidence of human spargana infections [1–5]. In 1882, the British doctor Manson identified spargana parasites in the subperitoneal fascia of a male corpse in Xiamen, China, and reported the first incidence of sparganosis in humans [6]. Since then, this disease has been found in 48 countries in Asia, Africa, Australia, Europe, and the Americas [7, 8]. Epidemiological studies reveal that sparganosis mostly affects East and Southeast Asia [9, 10], with China being a country affected by the pandemic, particularly in southwest and southeast China [11]. Thousands of cases of sparganosis have been reported globally, with the majority of cases happening in China, and the incidence of cerebral sparganosis is growing [12]. Spargana can induce a variety of symptoms depending on which tissues or organs are affected. Spargana primarily affects subcutaneous tissues, causing pain or urticaria, as well as the eyes, causing ocular sparganosis, which can lead to blindness. Sparganosis is commonly mistaken as a brain tumor in the central nervous system, and in extreme cases, it can cause convulsions, coma, paralysis, or death [6].

Infections with spargana are most commonly caused by ingesting raw or undercooked frog or snake meat, or drinking contaminated raw water. Fresh frog or snake flesh is used to treat skin ulcers and eye disorders in rural China. Different medical methods, such as drinking raw snake blood or consuming snake bile, are also used to treat different ailments [7]. Sparganosis is currently treated primarily through surgical excision of spargana. Surgery is appropriate for the treatment of subcutaneous, ophthalmic, and in some cases cerebral sparganosis, but only when the infection is in superficial regions of the brain and not in essential functional areas. Deep intracranial sparganosis has been treated with computed tomography (CT)-guided stereotactic aspiration, although relapses may occur [13]. As a result, there is currently no effective treatment for proliferative sparganosis or other sparganosis instances that cannot be surgically cured. Antiparasitic medications such as praziquantel, mebendazole, and albendazole are also ineffective in the treatment of sparganosis [14–16]. Albendazole prevents parasites from absorbing glucose by causing damage to intestines, progressively diminishing endogenous glycogen and causing a decrease in glycogen storage [17]. But it may also lead worms to die, because it inhibits fumaric acid reductase activity, lowers ATP synthesis, and alters the anaerobic metabolic pathway that worms utilize to obtain energy [18]. To disrupt energy metabolism in the parasite, praziquantel, a broad-spectrum antiparasitic medication, inhibits glucose absorption and increases lactic acid excretion [19]. In addition, pyrvinium embonate can disrupt the respiratory chain and the absorption of exogenous glucose by *Enterobius vermicularis*, depleting endogenous glycogen storage and ultimately leading to parasite death [20].

Almost all organisms rely primarily on glycolysis, the tricarboxylic acid cycle (TCA), gluconeogenesis, glycogenesis and glycogenolysis, and the pentose phosphate pathway (PPP) for energy. Instead of relying on a single glucose metabolic pathway, parasites rely on a combination of them. *Schistosoma mansoni* larvae parasitize *Biomphalaria glabrata* primarily through glycogenolysis, gluconeogenesis, and glycolysis pathways [21]. Although *Plasmodium* has a complete TCA cycle in red blood cells, it primarily relies on anaerobic glycolysis to obtain energy from glucose derived from the host for growth and reproduction [22]. Adult *Ascaris lumbricoides* worms get most of their energy from anaerobic glycolysis, acquiring glucose from the glycogenolysis route, but larvae get their energy from both glycolysis and the TCA cycle [23]. Furthermore, exogenous amino acids are crucial energy sources for *Clonorchis sinensis*, and they are used in gluconeogenesis to ensure adult worm survival in the host [24]. Wilms et al. discovered that taeniid tapeworms retain a substantial amount of glycogen in the tegument, which is generated from glucose received from the host [25].

Because intermediate and terminal hosts are necessary for the life cycle of *Spirometra mansoni* (*S. mansoni*), distinct patterns of carbohydrate metabolism must be adopted in order to adapt to abrupt changes in the environment, such as variations in temperature, ion concentrations, oxygen tension, and nutrient availability. Spargana typically parasitize the muscle tissues of frogs and snakes, which are difficult for them to survive in and obtain energy from because of the hypoxia, high protein and glycogen levels, and low-glucose environment. It is yet unknown, however, which nutrients spargana primarily uses, how the many routes involved in energy supply and metabolism of carbohydrates cooperate, and which pathways predominate during the development of spargana. Therefore, the purpose of this study was to identify the primary nutrients and define the dominant carbohydrate metabolic pathways in spargana’s energy source, which clarifies the pattern of carbohydrate metabolism and paves the way for further research in carbohydrate metabolism of other tapeworms. Our research integrates amino acid analysis, molecular biology detection, and transcriptome sequencing to replace old environmentally dangerous isotopic labeling methods, allowing us to develop a new strategy for studying organisms' energy metabolism. The findings can serve as an academic basis for the development of chemotherapeutic medicines against sparganosis, as well as having significant practical implications for solving clinical treatment challenges.

## Methods

### Animal and ethics statement

Wild frogs (*Fejervarya limnocharis* and *Pelophylax plancyi*) were anesthetized, and spargana were extracted from muscle tissue; next, the muscle tissue surrounding the spargana (0.5 cm × 0.5 cm × 0.5 cm) was excised (infected muscle tissue group). Noninfected muscle tissue (control tissue group) was obtained from a symmetrical section of the same frog. The Manipulative Technique for the Care and Use of Laboratory Animals (second revision) established by the State Scientific and Technological Commission of China was followed in all animal procedures. The Institutional Animal Care and Use Committee of Hainan University (Hainan, China) authorized the animal experimental protocols.

### Detection of free amino acids in *F. limnocharis* and *P. plancyi* muscle tissues

Multiple infected muscle tissues were combined into a single sample (around 1.0 g). There were six tissue samples detected in the *F. limnocharis* group, including three sparganum-infected and three noninfected tissue samples, and eight tissue samples detected in the *P. plancyi* group, including four sparganum-infected and four noninfected tissue samples. The samples were transferred to extraction tubes, 500 μl of 0.01 M HCl was added, and the samples were homogenized (5000 rpm, 20 s) with a tissue cell destroyer (DS1000, China). The homogenates were placed in 50-ml sterile centrifuge tubes and treated for 30 min at 25 °C with 5 ml of 0.01 mol/l HCl. The supernatants were collected and transferred into new sterile tubes after mixing and centrifugation, and an equal volume of 5% sulfonyl salicylic acid solution was added. The samples were properly mixed and incubated at 25 °C for 15 min. The supernatants were centrifuged and filtered through a 0.22 μm filter. Prior to amino acid analysis, the supernatants were lyophilized and mixed with 2 ml of sterile deionized water. Samples (10 μl) were applied to an amino acid analyzer (Sykam S 433D; Sykam GmbH, Eresing, Germany) under the following detection conditions: chromatographic column, Sykam LCA k07/Li; mobile phase, lithium citrate A = pH 2.90, B = pH 4.20, and C = pH 8.00; flow rate, 0.45 ml/min elution pump and 0.25 ml/min derivative pump; detection wavelength, 570 nm and 440 nm; and temperature 38–74 °C, gradient heating. The analyzer was used in accordance with the manufacturer's instructions. The concentration of free amino acids in each sample was calculated using the equation shown in Additional file 1: S1.

### Determination of glycogen, glucose, and glycerol concentrations in *P. plancyi* muscle tissues

Sparganum-infected and noninfected muscle tissues (about 40 mg) were homogenized in 400 μl of sterile deionized water for 10 s at 5000 rpm using a tissue cell destroyer. After boiling for 5 min to inactivate the enzymes, the samples were centrifuged at 12,000 rpm for 5 min to remove insoluble material. Fluorescence colorimetric assays were performed on the supernatants using a glycogen assay kit (Sigma-Aldrich, St. Louis, MO, USA). Glycogen concentration was determined using the manufacturer's protocol.

The muscle tissues (about 40 mg) were taken out using the same approach as described earlier. To obtain the supernatants, the samples were centrifuged at 12,000 rpm for 10 min. A glucose assay kit (Sigma-Aldrich) was used to measure glucose concentrations.

 Sparganum-infected and noninfected *P. plancyi* muscle tissues were weighed to 50 mg. The samples were placed in 400 μl of phosphate-buffered saline and extracted using the same technique as described previously. The supernatant was centrifuged, and 10 μl of each sample was transferred to separate wells of a 96-well plate. Glycerol concentrations were measured using a glycerol assay kit (Sigma-Aldrich) according to the manufacturer’s protocol.

### Analysis of transcriptional level of key enzymes involved in carbohydrate metabolism in the developmental stage of spargana

The sparganum larvae isolated from *P. plancyi* were gavage-infected in a 3-week-old cat, and adult worms were isolated from the cat's colon 3 months later. Adult worms were frozen in liquid nitrogen before being used for transcriptome sequencing. The transcriptome library of the adult worm *S. mansoni* was constructed in the laboratory of Dr. Gang Lu. The adult transcriptome library was screened for gene sequences of important enzymes involved in five carbohydrate metabolism pathways (TCA, glycolysis, gluconeogenesis, glycogenesis and glycogenolysis, and PPP) and used for primer design. Spargana were isolated from the muscles of *P. plancyi*, homogenized for total RNA extraction using the RNeasy Mini Kit (Qiagen, Hilden, Germany), and reverse-transcribed into complementary DNA (cDNA) using the RevertAid First Strand cDNA Synthesis Kit (Thermo Fisher Scientific, Waltham, MA, USA). The cDNA was used as a template for quantitative real-time polymerase chain reaction (PCR) amplification using the SYBR Premix Ex Taq kit (TaKaRa Bio, Shiga, Japan) on an Agilent Stratagene Mx3005P instrument (Agilent Technologies, Santa Clara, CA, USA). Primers for the genes encoding key enzymes are listed in Table 1. The PCR conditions were as follows: 95 °C for 30 s, 40 cycles of 95 °C for 5 s and 60 °C for 20 s. Data were analyzed by the 2^−ΔΔCT^ method [26].

**Table 1:** Primers used in quantitative real-time PCR

Gene name	Forward primer	Reverse primer
ATP citrate synthase (ATP-CS)	5′-GCAAAGCGACCCACTCTGATC-3′	5′-GCCAGTACAAAGAGCCCGTTAA-3′
Citrate synthase (CS)	5′-GTTCCTGGTTTCGGACACGC-3′	5′-GCACTGGGCGACAATCTTCA-3′
Isocitrate dehydrogenase (IDH)	5′-TCAGTCTTTATGCCAATGTGCG-3′	5′-GCCCGAGTATTCACCCTCTGTA-3′
α-Ketoglutarate dehydrogenase (α-KGDHC)	5′-ATCAAGGTTCCTGCCTTCCC-3′	5′-ATGGCGGTCTTGTCGGTTT-3′
Hexokinases (HK)	5′-TTGGACTAATTGTTGGCACCG-3′	5′-GATTCCCCAAAAGCACCCCAT-3′
6-Phosphofructokinase (PFK)	5′-TGGCGAATACATAAAGGAAGC-3′	5′-CACCACCAATGACGACAAGAG-3′
Pyruvate kinase (PK)	5′-CGGTATTATTATTCGCCGTGGTG-3′	5′-GTTTCATCCGCATGGTCTCAA-3′
Phosphoenolpyruvate carboxylase (PEPc)	5′-CCAACATGGCTATGCTGACCC-3′	5′-GACCCTCCCAGAAGAAACGAC-3′
Fructose bisphosphatase (FBP)	5′-GGCGAAGAGGTTAAGAAGTTGG-3′	5′-TATTTGCCCTGTTTATCGGGTT-3′
Glucose-6-phosphatase (G6Pase)	5′-TTTTCCACTTGCCTTCTTTCTTC-3′	5′-GTGTCAGGTGCAGTTTCTCCTTC-3′
Glycogen synthase (GS)	5′-AAAAGAGTCTGGGAGCGATGTG-3′	5′-ATTCACGGTCTCACGCAGTTGT-3′
Glycogen phosphorylase (GP)	5′-ATGTTCAATGACGACGGCTTTA-3′	5′-GTGACACCGTTGGTCTTATTCT-3′
Glucose-6-phosphate dehydrogenase (G-6-PD)	5′-TTTTCCACTTGCCTTCTTTCTTC-3′	5′-GTGTCAGGTGCAGTTTCTCCTTC-3′
6-Phosphogluconate dehydrogenase (6-PGDH)	5′-TGAAGCCTACCATCTTCTCCG-3′	5′-GACAGCCTCGTTCCAGACCAC-3′
*Spirometra mansoni* β-actin	5′-CATCTACGAGGGTTACGCACTG-3′	5′-GCTCATCTCCTGCTCAAAGTCC-3′

### Transcriptome analysis for spargana with different nutrient treatment and analysis of the effects on carbohydrate metabolism

Spargana were grown in 1 μg/ml glycogen, l-glutamine, low-glucose Dulbecco’s modified Eagle medium (DMEM; 1.0 g/l, containing l-glutamine), and high-glucose DMEM (4.5 g/l, containing l-glutamine) for 24 h at 5% CO_2_ and 37 °C, with media replaced every 12 h. There were eight spargana in each well and three in each group. Spargana grown in normal saline (NS) were used as the control group.

Spargana cultured under different nutrient conditions were sent to Gene Denovo Biotechnology Co., Ltd. (Guangzhou, China) for RNA extraction and identification, and sequenced using an Illumina NovaSeq 6000 (Illumina. Inc., San Diego, CA, USA). RNA differential expression analysis was performed between two different groups using DESeq2 software. Transcripts with a false discovery rate of less than 0.05 and an absolute fold change of less than 2 were designated as differentially expressed genes (DEGs). Detailed information is shown in Additional file 1: S2. The data were standardized and filtered using the gene expression array generated from the upstream high-throughput sequencing analysis. The R software package Mfuzz (v2.5, 8.0) was used for cluster analysis and visualization. Based on the screening, the cluster's specifically expressed genes were retained for further investigation.

Spargana samples cultured in glycogen, l-glutamine, low-glucose DMEM, and high-glucose DMEM were collected, homogenized for total RNA extraction, and reverse-transcribed into cDNA as described previously. The transcriptional levels of major enzymes involved in carbohydrate metabolism in spargana were examined using quantitative PCR. The experimental reactions were carried out, and the data analysis was performed in the same manner as described earlier.

### Statistical analysis

All experiments were carried out at least three times and the data were analyzed using SPSS software (version 13.0) and GraphPad Prism version 8.4.3 (686) software (La Jolla, CA, USA). The results are presented as the mean ± SD, and a *P*-value less than 0.05 was considered statistically significant.

## Results

### Comparison of free amino acids between sparganum-infected and noninfected muscles of *F. limnocharis* and *P. plancyi*

Thirty-one types of free amino acids were detected in the *F. limnocharis* group (Table 2 and Additional file 1: S2). The total concentrations of free amino acids in sparganum-infected and noninfected muscle tissues were not significantly different. The concentrations of 15 of them were significantly higher (*P* < 0.05) in infected muscle tissues than in noninfected muscle tissues. In the sparganum-infected muscle, the concentrations of glucogenic amino acids threonine (Thr), serine (Ser), asparagine (Asn), glutamic acid (Glu), glutamine (Gln), alanine (Ala), valine (Val), cystine (Cys), isoleucine (Ile), histidine (His), proline (Pro), glucogenic and ketogenic amino acids tyrosine (Tyr) and phenylalanine (Phe), phosphoethanolamine (PEA), and amino ethylenediamine increased by approximately 1.59-, 1.56-, 1.62-, 1.83-, 1.83-, 1.35-, 1.45-, 2.82-, 1.47-, 1.40-, 2.16-, 1.62-, 1.72-, 1.63-, and 3.06-fold, respectively, compared to those in noninfected muscle tissue.

**Table 2:** Comparison of free amino acids between sparganum-infected and noninfected muscle of *Rana limnocharis* (mg/ml, *N* = 3)

No.	Free amino acid	Noninfected muscle	Infected muscle	*P*-value
1	Phosphoserine (P-Ser)	5.08 ± 0.36	5.75 ± 1.03	n.s. (0.343)
2	Taurine (Tau)	15.33 ± 7.23	17.50 ± 7.31	n.s. (0.733)
3	Phosphorylethanolamine (PEA)	0.95 ± 0.24	1.55 ± 0.11	< 0.05 (0.017)
4	Urea (Ure)	374.06 ± 83.94	341.80 ± 73.31	n.s. (0.798)
5	Aspartic acid (Asp)^a^	5.87 ± 1.44	8.79 ± 1.16	n.s. (0.053)
6	Threonine (Thr)^a^	15.82 ± 1.81	25.14 ± 2.94	< 0.05 (0.009)
7	Serine (Ser)^a^	19.78 ± 2.41	30.86 ± 2.14	< 0.05 (0.004)
8	Asparagine (Asn)^a^	12.78 ± 0.67	20.76 ± 1.78	< 0.05 (0.012)
9	Glutamic acid (Glu)^a^	18.18 ± 2.38	33.20 ± 3.74	< 0.05 (0.004)
10	Glutamine (Gln)^a^	28.79 ± 2.62	52.65 ± 6.48	< 0.05 (0.004)
11	Amino ethylenediamine (α-AAA)	1.00 ± 0.42	3.06 ± 1.04	< 0.05 (0.033)
12	Glycine (Gly)^a^	19.06 ± 2.41	23.70 ± 5.46	n.s. (0.249)
13	Alanine (Ala)^a^	55.33 ± 1.72	74.50 ± 6.82	< 0.05 (0.009)
14	Aminobutyric acid (α-ABA)	0.56 ± 0.37	1.25 ± 0.62	n.s. (0.172)
15	Valine (Val)^a^	21.44 ± 2.13	31.07 ± 4.10	< 0.05 (0.022)
16	Cystine (Cys)^a^	1.36 ± 0.55	3.84 ± 1.33	< 0.05 (0.04)
17	Methionine (Met)^a^	12.84 ± 0.96	21.86 ± 6.56	n.s. (0.078)
18	Isoleucine (Ile)^a^	15.13 ± 1.18	22.25 ± 2.83	< 0.05(0.016)
19	Leucine (Leu)^c^	43.15 ± 4.43	73.12 ± 21.07	n.s. (0.073)
20	Tyrosine (Tyr)^b^	21.14 ± 3.02	34.31 ± 7.39	< 0.05 (0.046)
21	Phenylalanine (Phe)^b^	17.63 ± 3.52	30.26 ± 6.50	< 0.05 (0.042)
22	Alanine (β-Ala)	10.27 ± 0.69	16.17 ± 6.35	n.s. (0.185)
23	Aminoisobutyric acid (β-AiBA)	1.00 ± 0.49	6.88 ± 5.47	n.s. (0.137)
24	Aminobutyric acid (γ-ABA)	2.75 ± 0.50	5.84 ± 3.26	n.s. (0.181)
25	Histidine (His)^a^	15.29 ± 1.62	21.48 ± 2.44	< 0.05 (0.022)
26	Carnosine (Car)	924.93 ± 50.36	776.61 ± 116.52	n.s. (0.113)
27	Tryptophan (Trp)^b^	3.85 ± 2.81	1.54 ± 0.88	n.s. (0.360)
28	Ornithine (Orn)	4.93 ± 3.21	6.38 ± 2.22	n.s. (0.555)
29	Lysine (Lys)^c^	62.33 ± 18.10	80.85 ± 7.66	n.s. (0.160)
30	Arginine (Arg)^a^	37.40 ± 10.75	50.52 ± 10.58	n.s. (0.207)
31	Proline (Pro)^a^	7.83 ± 1.89	16.92 ± 1.44	< 0.05 (0.003)
32	Total amino acid	1775.62 ± 237.25	1837.16 ± 292.69	n.s. (0.791)

The data are presented as mean ± SD. Two-independent-sample *t*-test was used to evaluate the comparisons. *P* < 0.05 was considered as statistical significance

*n.s.* not significant

^a^Glucogenic amino acid

^b^Glucogenic and ketogenic amino acid

^c^Ketogenic amino acid

In the *P. plancyi* group, the total concentration of free amino acids in sparganum-infected muscle was 1.51-fold higher than that in noninfected muscle (*P* < 0.05), and 25 different species of free amino acids were detected (Table 3 and Additional file 1: S3). Additionally, the concentrations of the other 22 components of free amino acids in the sparganum-infected muscle of *P. plancyi* were significantly higher than those in noninfected muscle tissues (*P* < 0.05). In sparganum-infected muscles, the concentrations of glucogenic amino acids Asp, Thr, Ser, Asn, Glu, Gln, Gly, Ala, Val, Met, Ile, His, Arg, and Pro, glucogenic and ketogenic amino acids Tyr and Phe, and ketogenic amino acids leucine (Leu), lysine (Lys), phosphoserine (P-Ser), taurine (Tau), aminobutyric acid (*γ*-ABA), and ornithine (Orn) increased by approximately 2.54-, 3.02-, 3.43-, 4.08-, 3.00-, 3.98-, 2.26-, 2.84-, 2.60-, 2.72-, 2.65-, 12.43-, 2.99-, 3.11-, 2.98-, 3.13-, 3.14-, 2.95-, 1.70-, 1.91-, 3.64-, and 4.11-fold, respectively, compared to those in noninfected muscles.

**Table 3:** Comparison of free amino acids between sparganum-infected and normal muscle of *Rana plancyi* (mg/ml, *N* = 4)

No.	Free amino acid	Noninfected muscle	Infected muscle	*P*-value
1	Phosphoserine (P-Ser)	2.65 ± 0.18	4.5 ± 0.34	< 0.05 (0.001)
2	Taurine (Tau)	19.2 ± 3.64	36.62 ± 5.25	< 0.05 (0.037)
3	Urea (Ure)	246.99 ± 38.49	358.99 ± 57.63	n.s. (0.196)
4	Aspartic acid (Asp)^a^	4.81 ± 0.27	12.2 ± 0.36	< 0.05 (0.000)
5	Threonine (Thr)^a^	10.95 ± 1.24	33.06 ± 3.58	< 0.05 (0.000)
6	Serine (Ser)^a^	12.51 ± 1.72	42.95 ± 6.17	< 0.05 (0.001)
7	Asparagine (Asn)^a^	6.33 ± 0.52	25.8 ± 5.49	< 0.05 (0.007)
8	Glutamic acid (Glu)^a^	12.58 ± 1.26	37.71 ± 6.46	< 0.05 (0.004)
9	Glutamine (Gln)^a^	8.84 ± 0.67	35.17 ± 5.88	< 0.05(0.002)
10	Glycine (Gly)^a^	12.8 ± 1.45	28.87 ± 3.38	< 0.05 (0.007)
11	Alanine (Ala)^a^	24.89 ± 1.57	70.64 ± 9.32	< 0.05 (0.001)
12	Valine (Val)^a^	12.55 ± 1.41	32.62 ± 3.76	< 0.05 (0.001)
13	Methionine (Met)^a^	7.62 ± 0.90	20.7 ± 2.57	< 0.05 (0.001)
14	Isoleucine (Ile)^a^	11.14 ± 0.97	29.55 ± 3.01	< 0.05 (0.000)
15	Leucine (Leu)^c^	23.92 ± 2.45	75.12 ± 8.78	< 0.05 (0.000)
16	Tyrosine (Tyr)^b^	10.66 ± 1.10	31.72 ± 2.20	< 0.05(0.000)
17	Phenylalanine (Phe)^b^	9.98 ± 0.52	31.28 ± 3.56	< 0.05 (0.000)
18	Alanine (β-Ala)	9.5 ± 1.77	12.65 ± 0.29	n.s. (0.67)
19	Aminobutyric acid (γ-ABA)	0.14 ± 0.03	0.51 ± 0.11	< 0.05 (0.009)
20	Histidine (His)^a^	2.4 ± 0.81	29.84 ± 2.08	< 0.05 (0.000)
21	Carnosine (Car)	718.69 ± 21.47	726.25 ± 55.90	n.s. (0.814)
22	Ornithine (Orn)	2.40 ± 0.81	9.86 ± 1.78	< 0.05 (0.004)
23	Lysine (Lys)^c^	37.06 ± 2.35	109.22 ± 12.70	< 0.05 (0.000)
24	Arginine (Arg)^a^	20.00 ± 1.08	59.83 ± 6.27	< 0.05 (0.000)
25	Proline (Pro)^a^	8.00 ± 1.11	24.85 ± 2.22	< 0.05 (0.000)
26	Total amino acid	1247.94 ± 54.59	1889.85 ± 145.71	< 0.05 (0.003)

The data are presented as mean ± SD. Two-independent-samples test was used to evaluate the comparisons. *P* < 0.05 was considered as statistical significance

*n.s.* not significant

^a^Glucogenic amino acid

^b^Glucogenic and ketogenic amino acid

^c^Ketogenic amino acid

### Concentrations of glucose, glycogen, and glycerin in sparganum-infected and noninfected muscles of *P. plancyi*

**Fig. 1:**
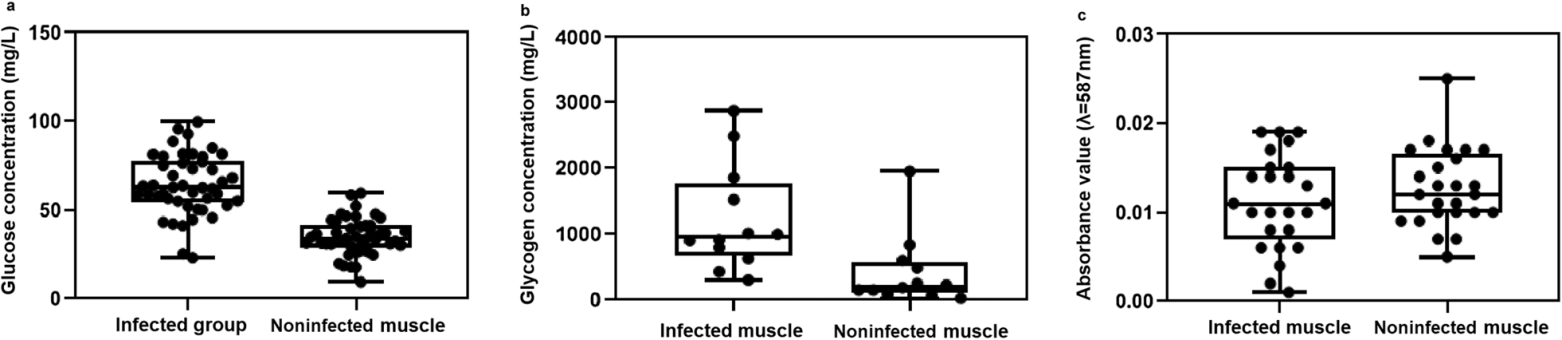
Detection of important nutrients in sparganum-infected and noninfected muscle of *Pelophylax plancyi*. **a** Glucose concentrations detected in sparganum-infected and noninfected *P. plancyi* muscle. **b** Glycogen concentrations detected in sparganum-infected and noninfected *P. plancyi* muscle. **c** Glycerol concentrations detected in sparganum-infected and noninfected *P. plancyi* muscle

The concentrations of glucose were approximately 1.15- to 5.32-fold higher in sparganum-infected tissues than in the noninfected tissues (*P* < 0.05) (Fig. 1a). The lowest glucose concentrations were 23.16 ± 0.4 and 17.70 ± 2.40 mg/l and the highest concentrations were 99.55 ± 4.20 and 59.69 ± 3.18 mg/l in the infected and noninfected muscle tissues, respectively.

The concentrations of glycogen in sparganum-infected muscle tissues was roughly 1.47- to 27.43-fold higher than in noninfected muscle tissues (Fig. 1b). In sparganum-infected and noninfected muscles, the lowest glycogen concentrations were 303.33 ± 23.9 and 29.8 ± 8.7 mg/l, while the maximum concentrations were 2876.1 ± 24.2 and 1950 ± 12.5 mg/l, respectively.

Glycerol was not detected in 16 samples of sparganum-infected and noninfected muscle tissues, respectively (Fig. 1c).

### Analysis of the transcriptional levels of key enzymes in carbohydrate metabolism pathways during spargana development

**Fig. 2:**
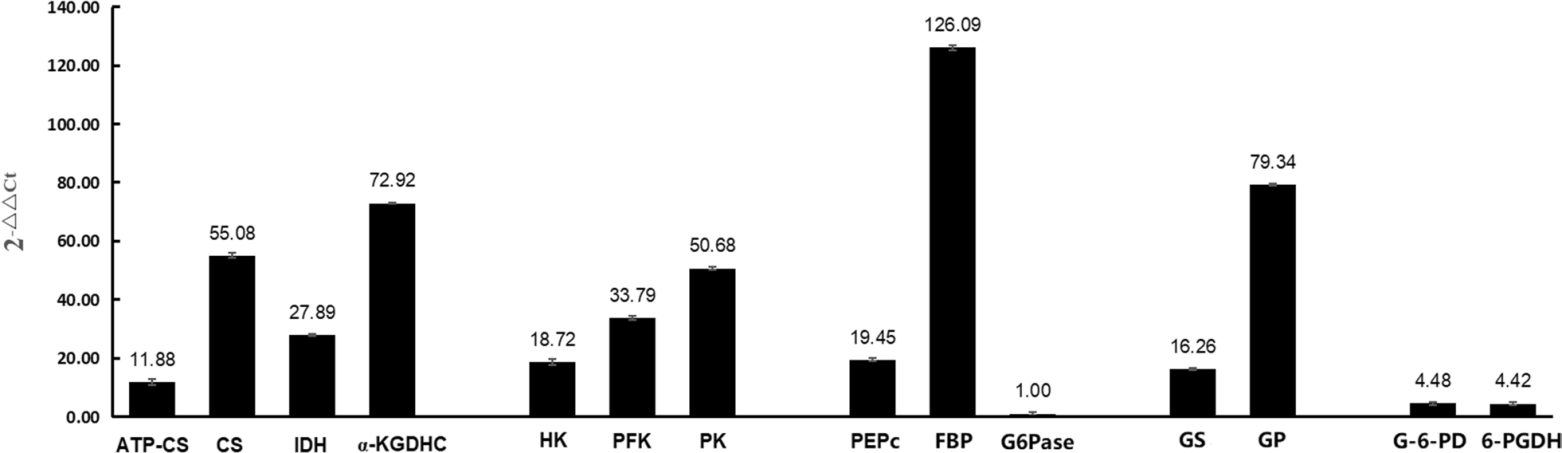
Analysis of the transcription levels of key enzymes in pathways of carbohydrate metabolism in spargana in vivo. *ATP-CS* ATP citrate synthase, *CS* citrate synthase, *IDH* isocitrate dehydrogenase, *α-KGDHC* *α*-ketoglutarate dehydrogenase complex, *HK* hexokinase, *PFK* 6-phosphofructokinase, *PK* pyruvate kinase, *PEPc* phosphoenolpyruvate carboxylase, *FBP* fructose biphosphatase, *G6Pase* glucose 6-phosphatase, *GS* glycogen synthase, *GP* glycogen phosphorylase, *G-6-PD* glucose-6-phosphate dehydrogenase, *6-PGDH* 6-phosphogluconate dehydrogenase

All enzymes identified in the five carbohydrate metabolic pathways were transcribed (Fig. 2). The transcriptional level of glucose-6-phosphatase (G6Pase) was the lowest of all the enzymes found in the gluconeogenesis pathway, whereas the transcriptional level of the rate-limiting enzyme fructose bisphosphatase (FBP) was the highest (about 126.09-fold higher than that of G6Pase). The transcriptional level of glycogen phosphorylase (GP) in the glycogenolysis pathway was 4.88-fold higher than that of glycogen synthase (GS) in the glycogenesis pathway.

### Transcriptome sequencing analysis of spargana under different nutrient conditions

**Fig. 3:**
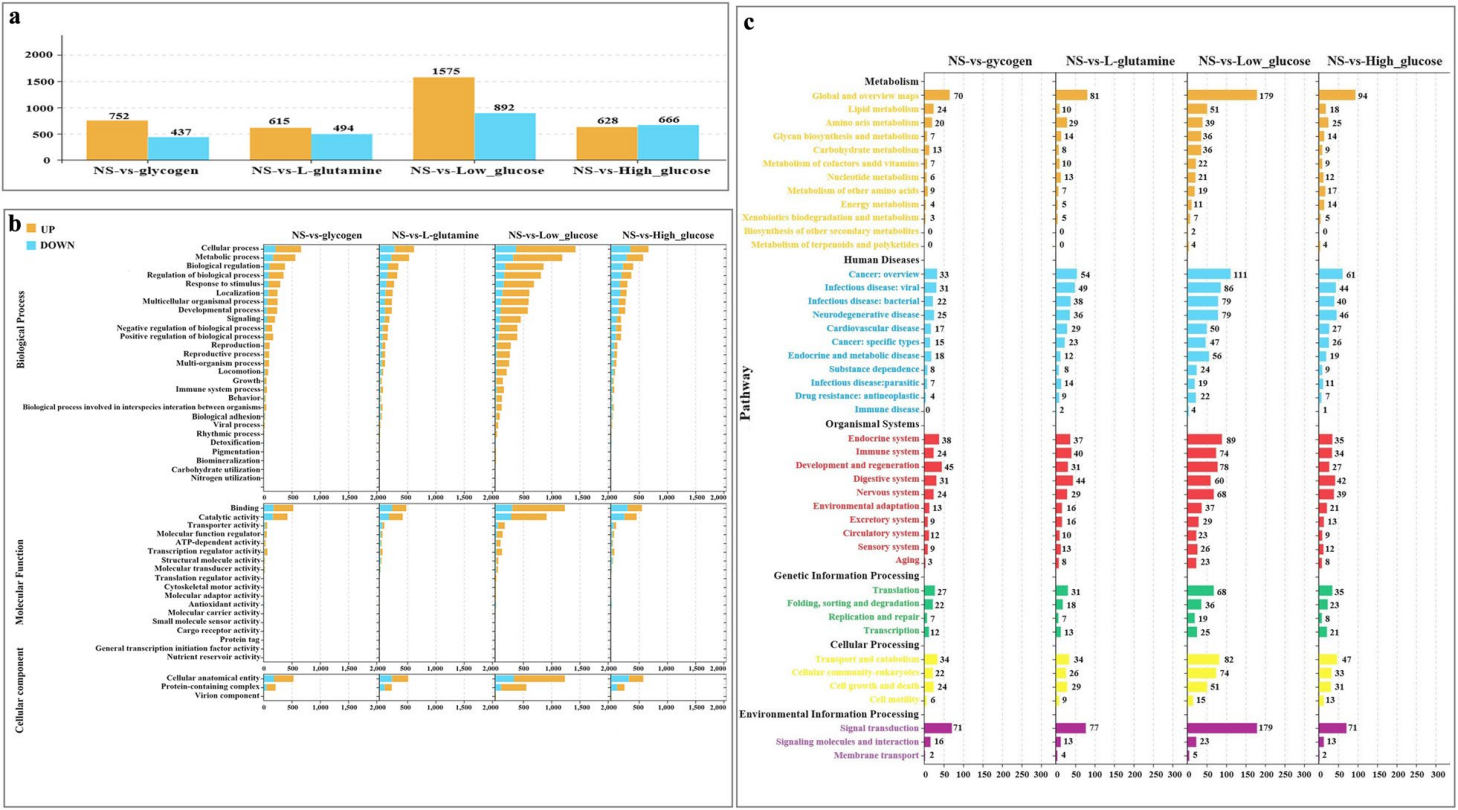
Analysis of differentially expressed genes (DEGs) in different nutritional groups. **a** DEGs in different nutritional groups. **b** GO analysis of DEGs in different nutrient groups. **c** The KEGG analysis of DEGs in different nutrient groups. In a and b, the upregulated DEGs are orange, while the downregulated DEGs are blue. The pathways involved in metabolism are colored orange; the pathways involved in human diseases are colored blue; the pathways involved in organismal systems are colored red; the pathways involved in genetic information processing are colored green; the pathways involved in cellular processes are colored yellow; and the pathways involved in environmental information processing are colored purple

The transcriptome data have been uploaded to the database (https://ngdc.cncb.ac.cn/gsa/), with accession number CRA014115. According to transcriptome analysis, the low-glucose group had the highest number of DEGs (DEGs), with 1575 up- and 892 downregulated genes (Fig. 3a). The functional domains of DEGs in the three categories for different nutritional groups were almost equivalent in Gene Ontology (GO) analysis (Fig. 3b). The number of DEGs was significantly higher in the low-glucose group in GO and Kyoto Encyclopedia of Genes and Genomes (KEGG) pathway analysis, and the primary pattern of these DEGs was upregulation (Fig. 3c).

**Fig. 4:**
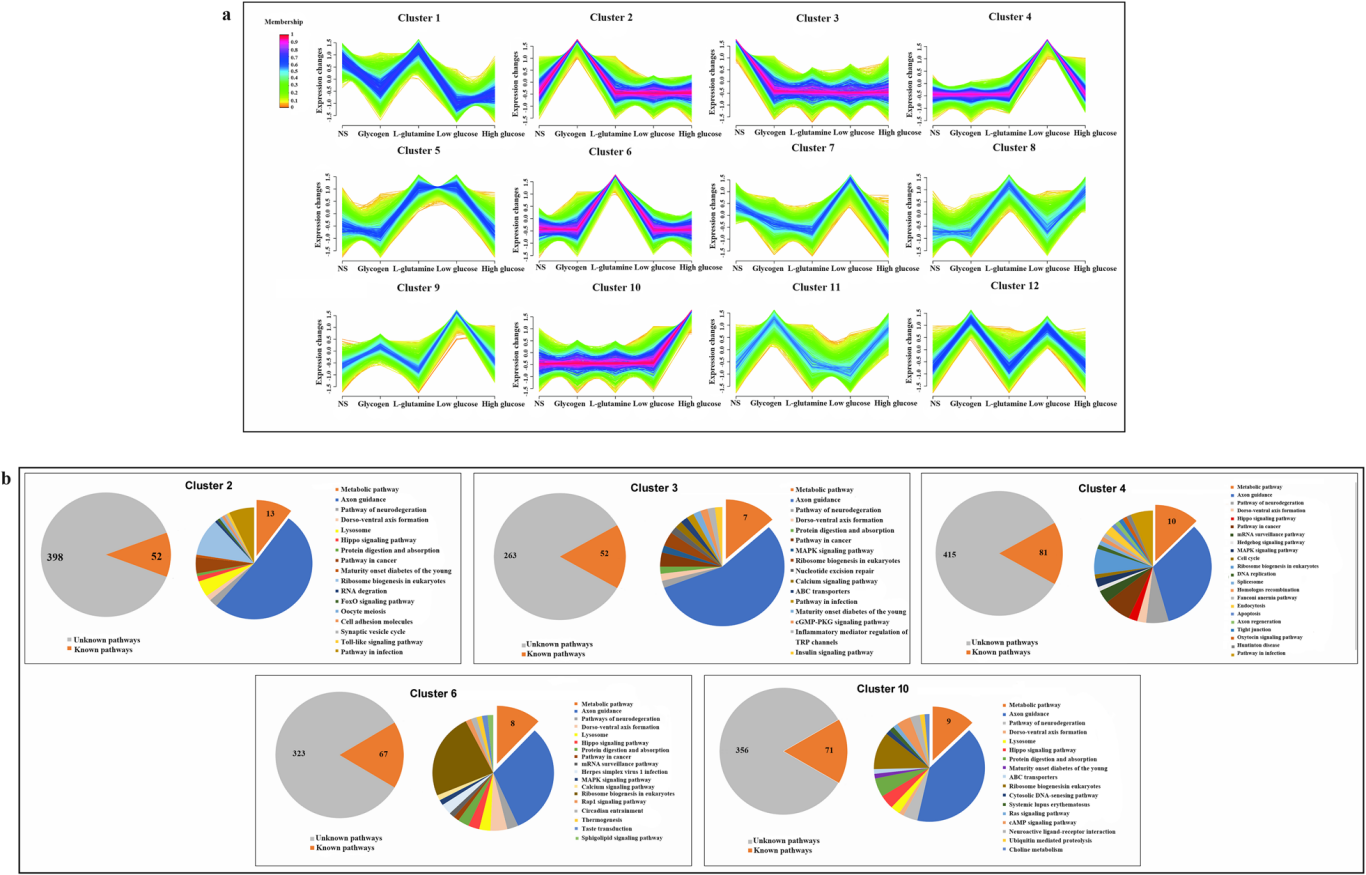
Screening and annotation of differentially expressed genes (DEGs). **a** Screening of DEGs in different nutrient treatment groups. Cluster 2 only contains DEGs in the glycogen treatment group. DEGs in the NS treatment group are shown in Cluster 3. Cluster 4 solely contains DEGs in the low-glucose therapy group. Cluster 6 solely contains DEGs in the l-glutamine therapy group. Cluster 10 only has DEGs in the high-glucose treatment group. **b** The number and functional distribution of DEGs in the five clusters

We performed cluster analysis and obtained clusters 2, 3, 4, 6, and 10, which were screened for genes solely expressed in the glycogen, NS, low-glucose, l-glutamine, and high-glucose groups (Fig. 4a). The number and functional distribution of DEGs in the five clusters are illustrated in Fig. 4b.

STRING analysis was used to identify the DEGs participating in the metabolic pathways in the clusters presented in Table 4, as well as the interaction diagrams.

**Table 4:** DEGs involved in metabolism in the KEGG pathway

Cluster	Group	DEGs involved
Cluster 2	Glycogen	Hexokinase β-1,3-galactosyltransferase Enolase Glycosyltransferases Adenylate cyclase 9 Myophosphorylase Dihydrolipoamide S-acetyltransferase Aldehyde dehydrogenase
Cluster 3	NS	γ-Glutamyltransferase 1 Carbonic anhydrase 14 Guanylate cyclase 2F S-Adenosylmethionine decarboxylase
Cluster 4	Low-glucose	Inositol polyphosphate 5 phosphatase Histidine decarboxylase Glucoside xylosyltransferase 1 Lysophospholipid acyltransferase 1-like 5′-Nucleotidase LISK family protein kinase Guanylate cyclase 2D
Cluster 6	l-Glutamine	α-1,6-Fucosyltransferase Glycoprotein-n-acetylgalactosamine 3-β-galactosyltransferase 1 Adenylate kinase 2 Nudix hydrolase 9 NADH:ubiquinone oxidoreductase 51kD subunit Leucine aminopeptidase 2
Cluster 10	High-glucose	Lactate dehydrogenase B Galactosylxylosylprotein 3-β-glucuronosyltransferase I Glutamate dehydrogenase 1 Pyruvate carboxylase Ceramide synthase 1 Alkaline phosphatase α-Glucosidase

**Fig. 5:**
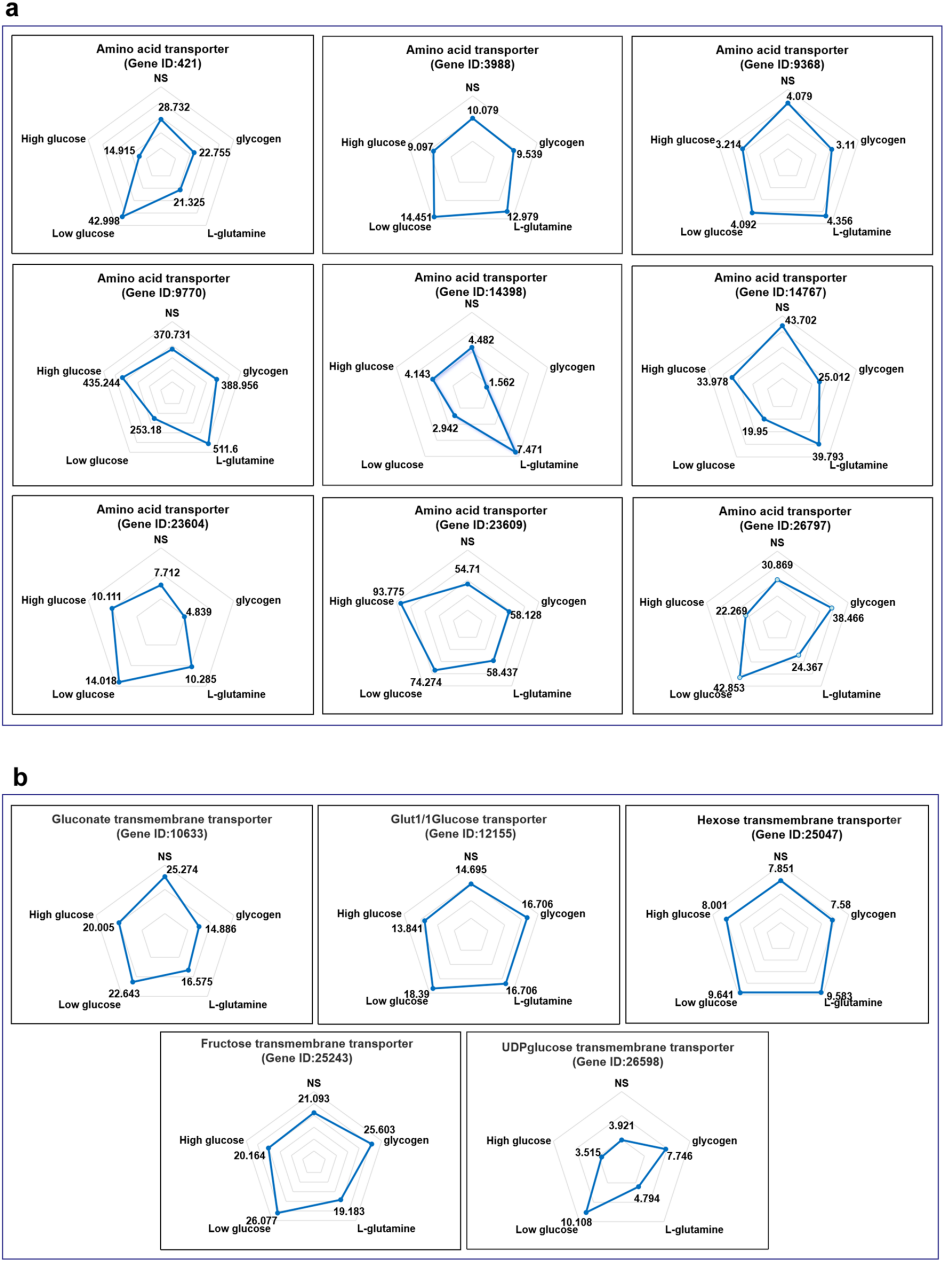
Fragments per kilobase per million mapped fragments (FPKM) values of different genes **a** The FPKM values for genes encoding amino acid transporters in the transcriptome analysis of the different nutrient treatment groups. **b** FPKM value of genes encoding carbohydrate transporters in the transcriptome analysis of different nutrient treatment groups. NS: spargana cultivated in normal saline (0.9%); glycogen: spargana cultivated in 1 mg/l glycogen; l-glutamine: spargana cultivated in DMEM containing l-glutamine (no glucose DMEM); low glucose: spargana cultivated in DMEM containing l-glutamine with glucose (1.0 g/l); high glucose: spargana cultivated in DMEM containing l-glutamine with glucose (4.5 g/l)

**Fig. 6:**
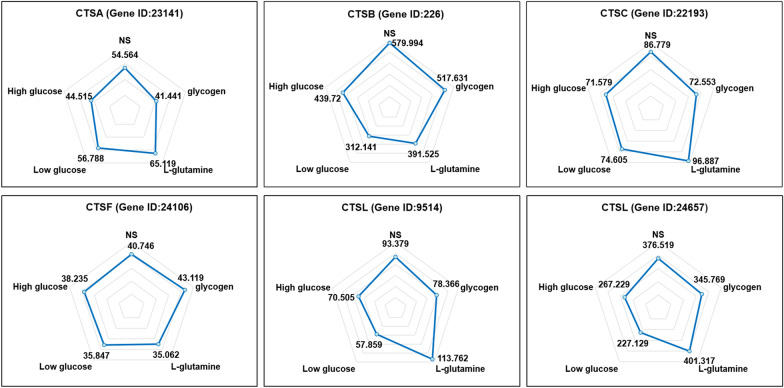
Fragments per kilobase per million mapped fragments (FPKM) values of the genes encoding cathepsins in transcriptome analysis of different nutrient culturing groups. NS: spargana cultivated with normal saline (0.9%); glycogen: spargana cultivated in 1 mg/l glycogen; l-glutamine: spargana cultivated in DMEM containing l-glutamine (no glucose DMEM); low glucose: spargana cultivated in DMEM containing l-glutamine with glucose (1.0 g/l); high glucose: spargana cultivated in DMEM containing l-glutamine with high glucose (4.5 g/l)

We discovered that nine genes encoding amino acid transporters and the FPKM (fragments per kilobase per million mapped fragments) values of genes (gene ID: 421, 14398, 14767, 23604, and 26797) in the l-glutamine and low-glucose groups were higher than those in the other groups (Fig. 5a). Gluconate transmembrane transporter, glut1/1 glucose transporter, hexose transmembrane transporter, fructose transmembrane transporter, and uridine diphosphate (UDP) glucose transmembrane transporter were the five carbohydrate transporter-encoding genes (Fig. 5b). Only the FPKM value of the UDP glucose transmembrane transporter changed, and increased in the low-glucose and glycogen groups. Furthermore, we discovered genes encoding members of the cathepsin family (*CTSA, CTSB, CTSC, CTSF, and CTSL*) (Fig. 6).

### Effects of different nutrients on the carbohydrate metabolism of spargana

**Fig. 7:**
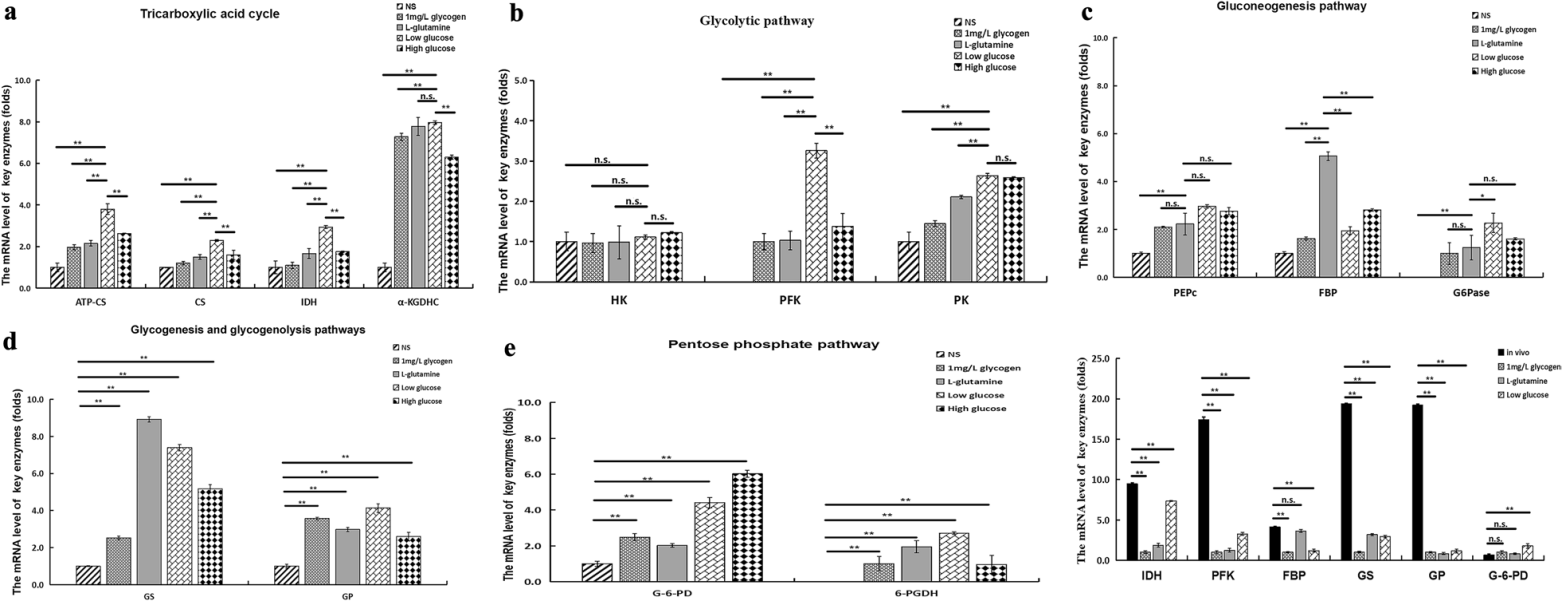
Effects of different nutrients on carbohydrate metabolism of spargana. **a** The messenger RNA (mRNA) levels of enzymes in the tricarboxylic acid cycle. **b** The mRNA levels of enzymes in the glycolytic pathway. **c** The mRNA levels of enzymes in the gluconeogenesis pathway. **d** The mRNA levels of enzymes in the glycogenesis and glycogenolysis pathways. **e** The mRNA levels of enzymes in the pentose phosphate pathway. **f** Comparison of carbohydrate enzyme transcription levels between in vivo and in vitro experimental conditions. **P < 0.05, analyzed by one-way analysis of variance (ANOVA), followed by the modified Tukey test for multiple comparisons, *n* = 6; *n.s.* not significant

The transcriptional levels of key TCA cycle enzymes were highest in the low-glucose group and lowest in the NS group. The transcriptional levels of ATP citrate synthase (ATP-CS), citrate synthase (CS), isocitrate dehydrogenase (IDH), and alpha-ketoglutarate dehydrogenase (α-KGDHC) were 3.80-, 2.30-, 2.94-, and 7.96-fold higher in the low-glucose group than in the NS group (Fig. 7a).

Except for the NS group, the transcriptional level of the major rate-limiting enzyme 6-phosphofructokinase (PFK) in the glycolytic pathway was detected in all groups. The low-glucose group had the highest transcriptional levels of the key enzymes (PFK and pyruvate kinase [PK]) (Fig. 7b).

G6Pase was not detected in the NS group. Furthermore, the transcriptional level of FBP was highest in the l-glutamine group (5.06-fold higher than in the NS group) and statistically different from the other groups (Fig. 7c).

GS and GP transcripts were detected in the glycogenesis and glycogenolysis pathways in all five nutritional culture groups. When compared to the NS group, the transcriptional level of GS was highest in the l-glutamine group (8.92-fold), followed by the low-glucose group (7.40-fold), high-glucose group (5.18-fold), and glycogen group (2.53-fold). The transcriptional levels of GP were 3.56-, 2.98-, 4.14-, and 2.60-fold higher in the glycogen, l-glutamine, low-glucose, and high-glucose groups, respectively, than that in the NS group. Notably, the transcriptional level of GP was 1.59-fold higher in the low-glucose group than in the high-glucose group (*P* < 0.05, Fig. 7d).

Glucose-6-phosphate dehydrogenase (G-6-PD) transcripts of the PPP were not detected in the NS group. However, transcripts of both enzymes were found in the other four groups (Fig. 7e).

The transcriptional levels of critical rate-limiting enzymes in five carbohydrate metabolic pathways were compared in spargana grown in *P. plancyi* muscle to spargana grown in distinct nutritional groups (glycogen, l-glutamine, and low-glucose). The transcriptional levels of IDH, PFK, GS, and GP were highest in spargana grown in *P. plancyi* muscle (Fig. 7f). G-6-PD expression in the PPP was lowest in spargana grown in *P. plancyi* muscle and highest (2.68-fold) in the low-glucose group.

## Discussion

The growth and development of parasites spans a variety of intermediate hosts, residing in various tissues and organs with vastly varying nutritional conditions. As a result, the host, tissue involvement, and local nutritional environment all have an impact on the parasite's nutrient sources and carbohydrate metabolism. Parasites must adapt to a variety of environmental variables, including oxygen, temperature, and nutrient availability. As an energy source, adult schistosomes in the host bloodstream import glucose across their tegument [27]. Exogenous amino acids may be required for adult *C. sinensis* survival in bile ducts [24]. The intestinal site influences worm germinal development in dogs infected with *Echinococcus granulosus*, and different adult worms rely upon different energy metabolism pathways [28]. Although energy supply is critical for spargana, nutritional sources and carbohydrate metabolic mechanisms remain unknown.

We discovered that the species and amounts of free amino acids differed between *F. limnocharis* and *P. plancyi* in sparganum-infected muscle. The absence of six free amino acids in *P. plancyi* may be attributable to the varied detection limits of the two analytical techniques. Furthermore, the total concentration of free amino acids in *F. limnocharis* was significantly (1.42-fold) higher than that in *P. plancyi*, which may be attributed to significant increases in urea and carnosine concentrations in *F. limnocharis* noninfected muscles by 1.52- and 1.29-fold, respectively, compared to those levels in *P. plancyi*. The total amount of free amino acids in sparganum-infected muscles did not differ between *F. limnocharis* and *P. plancyi* groups. Although the concentrations of free amino acids in noninfected muscle of different species of frogs varies, after infection by spargana, they increased, particularly those involved in energy metabolism (e.g., glucogenic amino acids).

Several prior investigations have indicated amino acids as the primary source of nutrition for parasites. *Toxoplasma gondii*, for example, requires amino acids for feeding [29]. Cysticerci prefer to absorb free amino acids from the host environment [30], while free amino acids have been identified in hydatid cyst fluids [31]. Tapeworms have a very limited biosynthetic metabolism and must rely on their hosts to acquire sugars, most amino acids, nucleosides, and fatty acids to synthesize their own macromolecules [32]. Furthermore, *E. granulosus* protoscoleces can accumulate l-amino acids and utilize multiple mechanisms for amino acid absorption through the tegument [33, 34]. The existence of coding genes for amino acid transporters was discovered in tapeworm genomes as well [32]. Transcriptome sequencing was also utilized to detect nine distinct genes encoding amino acid transporters. It was suggested that amino acid transporters in the sparganum tegument transport free amino acids from the host environment into the parasite, and that glucogenic and ketogenic amino acids are major contributors to carbohydrate metabolic pathways involved in cellular energy production. Spargana can use other amino acids for protein synthesis, neurotransmission, and other processes vital to their growth and development. Ketogenic amino acids (Leu and Lys) were shown to be elevated in sparganum-infected muscles, implying that ketone bodies could be an alternative energy source; however, further investigation is needed. Pro has been found to be raised in sparganum-infected muscles, which is critical in the maintenance of healthy skin and cloven-hoof tissues, especially wound tissues [35]. The discovery implies that Pro is released from host cells in response to chronic inflammation caused by mechanical injury and chemical stimulation by spargana.

The amino acid sources in the host environment can be created in the following ways. In the first place, significant inflammation develops around the sparganum-infected muscle in frogs [36]. Transcriptome sequencing research revealed that a number of genes encoding cathepsins were expressed in spargana in our investigation. Parasites may release numerous cathepsins to hydrolyze host proteins, which may be protective against host elimination and result in the production of many free amino acids [37–39]. Furthermore, as spargana moves and absorbs nutrition, it constantly rubs against muscle tissues and small veins, causing tissue injury and blood vessel rupture. As a result, many amino acids derived from plasma, such as Ser, Ala, and His, are markedly elevated in sparganum-infected muscles.

In addition, glucose and glycogen concentrations increased in the area around sparganum-infected muscles, and five genes encoding carbohydrate membrane transporters were discovered. Glucose transporters 1 and 4 in the tegument of mature schistosomes can import exogenous glucose into the parasite [27]. Glycogen is a branched polymer of glucose that serves as a reservoir of glucose, and the most significant site for glycogen storage is skeletal muscle. The glucose required for parasites is generally believed to be supplied by the destruction of host glycogen; for example, *Trichomonas vaginalis* secretes enzymes that hydrolyze host glycogen, releasing glucose [40, 41].

DEGs involved in metabolism were screened in the glycogen culturing group, including glycosyltransferases, adenylate cyclase 9, and myophosphorylase. Our findings demonstrate that muscle glycogen is digested into glucose and delivered into spargana via glucose transporters, implying that glucose supplies found in *P. plancyi* muscles are adequate to support spargana growth and development.

We also discovered that spargana has a comprehensive carbohydrate metabolic route for energy supply in vivo. Furthermore, in GO and KEGG analyses, the functional domains of DEGs in biological process categories and metabolic pathways for different nutritional groups were almost identical. However, there were significant differences in the number of DEGs, with the low-glucose group having more than the other groups, and it was discovered that after culturing spargana for 24 h in a low-glucose medium, the transcriptional levels of enzymes involved in the five carbohydrate metabolism pathways increased, indicating that spargana are more metabolically active in this nutritional environment than in others. Spargana cultivated in high concentration of glucose (high-glucose group) did not promote rapid energy generation, implying that the high concentration of glucose was an adverse environment for spargana. Notably, only key enzymes of the TCA, glycogenesis, and glycogenolysis pathways were expressed in the NS group, indicating that in the absence of nutrients, the gluconeogenesis pathway cannot convert glucose, and both the PPP and glycolysis pathway are blocked and unable to provide energy supply for spargana. This organism is well known for its ability to build glycogen and mobilize glycogen reserves in response to carbohydrate restrictions, a system that is crucial for meeting its energy requirements [42]. The outer layer of the embryonic envelope in the cestode *Echinococcus multilocularis* remains metabolically active, implicated in glycogen storage for development and survival [43]. Parasites have a high capacity for nutrient assimilation; for example, cysticerci absorb and consume large amounts of glucose via glucose transporters, and store extra glycogen for emergency consumption [44]. As a result, spargana may hydrolyze their limited glycogen stores for energy production in the short term, as needed, via the action of GP and subsequent metabolism in the TCA cycle, or conversely, augment glycogen storage via the glycogenesis pathway. In summary, spargana depends on the TCA, glycogenesis, and glycogenolysis pathways when it faces severe nutritional scarcity, such as during invasion and migration. Our findings imply that the major rate-limiting enzymes (IDH, GS, and GP) implicated in these pathways are promising targets for medication, which may be particularly significant if applied early in the course of treating sparganosis. Furthermore, we discovered that, of all the groups, the glucogenic amino acid (l-glutamine) group had the greatest transcriptional levels of the rate-limiting enzyme (FBP) in gluconeogenesis and GS in glycogenesis. This outcome was attributed to the fact that gluconeogenesis and glycogen synthesis are started for energy supply and nutrition transformation when only amino acids are present. Interestingly, the transcriptional levels of GP were highest in the glycogen and low-glucose groups, indicating that glycogenolysis is constantly triggered to maintain an appropriate glucose environment regardless of the presence of glucose. The primary rate-limiting enzyme in the glycolytic pathway and the transcriptional level of 6-PGDH in the PPP were not detected in the NS group. Reductive coenzyme II (NADPH) is needed to maintain redox dynamic equilibrium. The PPP is almost universal and metabolizes the glycolytic intermediates fructose 6-phosphate and glyceraldehyde 3-phosphate to produce ribose 5-phosphate, which is needed for the biosynthesis of nucleic acids, sugar-phosphate precursors, and amino acid biosynthesis [45]. These findings suggest a synergistic relationship between these two metabolic pathways. In other words, when one pathway is obstructed, it also impacts the other pathway. In conclusion, the NS environment (poor energy metabolism efficiency), which is detrimental to spargana survival, is unaffected by the PPP’s antioxidant effects. Nonetheless, the NS culture group’s transcriptome analysis showed that the DEG γ-glutamyltransferase 1 is responsible for the production of glutathione, the main intracellular antioxidant defense against oxidative stress. These results suggest that adaptations of the organism’s carbohydrate-metabolizing pathways may occur in response to nutrient variations. In other words, there is a close relationship between nutritional substrates and the use of several metabolic pathways in spargana.

Furthermore, with the exception of the PPP, we discovered that the transcriptional levels of the main rate-limiting enzymes in each of the carbohydrate pathways were much higher in vivo than in mono-nutrient groups. The PPP is a critical component of cellular metabolism, serving as a precursor for nucleotide and amino acid biosynthesis, reducing molecules for anabolism, and preventing oxidative stress [46]. The essential PPP enzymes were found at lower levels in spargana grown in vivo, which could be related to the fact that the spargana and host are well accustomed to one another, reducing the effect of oxidative stress. These findings imply that spargana's nutrient metabolic environment is multi-nutritional, and that nutrient use and metabolic patterns are more complex than expected. This conclusion is compatible with the nutrients found in sparganum-infected frog muscles and the transcription-level study of major enzymes involved in spargana carbohydrate metabolism in vivo. Additionally, we discovered that the transcriptional level of FBP in the gluconeogenic pathway was as high in spargana in vivo as it was in the l-glutamine group. This finding suggests that spargana require glucogenic amino acids to survive in the presence of a variety of nutrients in the host, and that gluconeogenesis is also required for spargana energy metabolism.

## Conclusions

The main nutritional sources of spargana in *F. limnocharis* and *P. plancyi* muscles are exogenous free amino acids, glycogen, and glucose obtained from the host via amino acid and glucose transporters, and glucogenic and/or ketogenic amino acids are converted into directly usable glucose through gluconeogenesis, and the worms' coordinated carbohydrate metabolism pathways use this glucose to generate energy. Spargana has a variety of nutritional sources, and there is a close relationship between nutrients and the carbohydrate metabolism pathways. Further, the TCA, glycogenesis, and glycogenolysis pathways are critical for spargana survival and development during invasion and migration. Gluconeogenesis is an indispensable metabolic mechanism in spargana growth and development. This study provides an important theoretical basis for future chemotherapeutic drug development for sparganosis and is significant in alleviating therapeutic challenges.

### Supplementary Information

**Additional file 1: S1.** Equations for calculation of the concentration of free amino acids in each. **S2.** The procedure of the transcriptome analysis applied in the study. **S3.** HPLC analytic profiles of amino acids in *Fejervarya limnocharis*. **S4.** HPLC analytic profiles of amino acids in *Pelophylax plancyi*. **S5.** STRING interaction diagram. PYGB: Myophosphorylase; ADCY9: Adenylate cyclase 9; GGT1: γ-glutamyltransferase 1; Amd2: S-adenosylmethionine decarboxylase; HDC :Histidine decarboxylase; GXYLT1: Glucoside xylosyltransferase 11; LAP2: Leucine aminopeptidase 2; AK2: Adenylate kinase 2; Ldhb: Lactate dehydrogenase B; GLUD1: Glutamate dehydrogenase 1.

## Data Availability

The transcriptome data are available in the database - https://ngdc.cncb.ac.cn/gsa/, with the accession number CRA014115. The article and/or supplementary materials contain all other data supporting the study's findings.
