# Supplementary material for: Important nutrient sources and carbohydrate metabolism patterns in the growth and development of spargana
Source: Parasit Vectors. 2024 Feb 16;17:68. doi: 10.1186/s13071-024-06148-1 (PMC10873960; doi:10.1186/s13071-024-06148-1)
Supplement: Supplementary file 1 — Additional file 1: S1. Equations for calculation of the concentration of free amino acids in each. S2. The procedure of the transcriptome analysis applied in the study. S3. HPLC analytic profiles of amino acids in Fejervarya limnocharis. S4. HPLC analytic profiles of amino acids in Pelophylax plancyi. S5. STRING interaction diagram. PYGB: Myophosphorylase; ADCY9: Adenylate cyclase 9; GGT1: γ-glutamyltransferase 1; Amd2: S-adenosylmethionine decarboxylase; HDC :Histidine decarboxylase; GXYLT1: Glucoside xylosyltransferase 11; LAP2: Leucine aminopeptidase 2; AK2: Adenylate kinase 2; Ldhb: Lactate dehydrogenase B; GLUD1: Glutamate dehydrogenase 1. [file 13071_2024_6148_MOESM1_ESM.zip › Supplementary material/S 1. The concentration of free amino acids in each sample was calculated using the equation.docx]

1.The concentration of free amino acids in each sample was calculated using the [equation](file:///C:/Users/Dell/AppData/Local/youdao/dict/Application/8.9.3.0/resultui/html/index.html#/javascript:;).


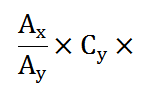
Initial concentrations:C_x_= F
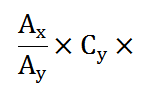


Amount(mg)= F×V

C_x_---The concentrations of amino acid in the sample was determined, mg/ml.


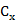


C_y_---The concentrations of amino acid in the standard, mg/ml. F---Sample dilution folds.

V---Volume of sample, ml.

A_x_---The peak area of the sample was determined.

A_y_---Peak area of amino acids in the standard.
