# Supplementary material for: Important nutrient sources and carbohydrate metabolism patterns in the growth and development of spargana
Source: Parasit Vectors. 2024 Feb 16;17:68. doi: 10.1186/s13071-024-06148-1 (PMC10873960; doi:10.1186/s13071-024-06148-1)
Supplement: Supplementary file 1 — Additional file 1: S1. Equations for calculation of the concentration of free amino acids in each. S2. The procedure of the transcriptome analysis applied in the study. S3. HPLC analytic profiles of amino acids in Fejervarya limnocharis. S4. HPLC analytic profiles of amino acids in Pelophylax plancyi. S5. STRING interaction diagram. PYGB: Myophosphorylase; ADCY9: Adenylate cyclase 9; GGT1: γ-glutamyltransferase 1; Amd2: S-adenosylmethionine decarboxylase; HDC :Histidine decarboxylase; GXYLT1: Glucoside xylosyltransferase 11; LAP2: Leucine aminopeptidase 2; AK2: Adenylate kinase 2; Ldhb: Lactate dehydrogenase B; GLUD1: Glutamate dehydrogenase 1. [file 13071_2024_6148_MOESM1_ESM.zip › Supplementary material/S 2 . Transcriptome analysis for spargana.pdf]

1. Flow chart of Experiments

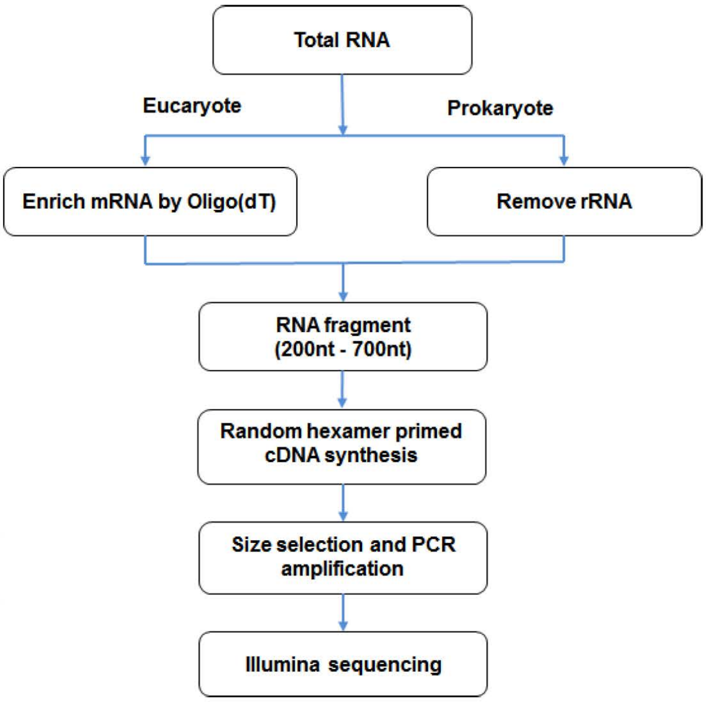

Fig 1. Flow chart of transcriptome experiments

1.2 RNA Extraction, library construction and sequencing

Total RNA was extracted using Trizol reagent kit (Invitrogen, Carlsbad, CA,USA) according to the manufacturer's protocol. RNA quality was assessed on an Agilent 2100 Bioanalyzer (Agilent Technologies, Palo Alto, CA, USA) and checked using RNase free agarose gel electrophoresis. After total RNA was extracted, eukaryotic mRNA was enriched by Oligo(dT) beads（原核：After total RNA was extracted, prokaryotic mRNA was enriched by removing rRNA by Ribo-ZeroTM Magnetic Kit (Epicentre, Madison, WI, USA)). Then the enriched mRNA was fragmented into short fragments using fragmentation buffer and reversly transcribed into cDNA by using NEBNext Ultra RNA Library Prep Kit for Illumina(NEB #7530,New England Biolabs, Ipswich, MA, USA). The purified double-stranded cDNA fragments were end repaired, A base added, and ligated to Illumina sequencing adapters. The ligation reaction was purified with the AMPure XP Beads(1.0X). And polymerase chain reaction (PCR) amplified. The resulting cDNA library was sequenced using Illumina Novaseq6000 by Gene Denovo Biotechnology Co. (Guangzhou, China).

2. Bioinformatics analysis

2.1 Filtering of Clean Reads

Reads obtained from the sequencing machines includes raw reads containing adapters or low quality bases which will affect the following assembly and analysis. Thus, to get high quality clean reads, reads were further filtered by fastp [1] (version 0.18.0). The parameters were as

follows:

1) removing reads containing adapters;

2) removing reads containing more than 10% of unknown nucleotides(N);

3) removing low quality reads containing more than 50% of low quality (Q-value≤20) bases.

2.2 Alignment with Ribosome RNA (rRNA)

Short reads alignment tool Bowtie2 [2] (version 2.2.8) was used for mapping reads to ribosome RNA (rRNA) database. The rRNA mapped reads then will be removed. The remaining clean reads were further used in assembly and gene abundance calculation.

2.3 Alignment with Reference Genome

An index of the reference genome was built, and paired-end clean reads were mapped to the reference genome using HISAT2. 2.4[3] and other parameters set as a default.

2.4 Quantification of Gene Abundance

The mapped reads of each sample were assembled by using StringTie v1.3.1[4][5] in a reference-based approach. For each transcription region, a FPKM (fragment per kilobase of transcript per million mapped reads) value was calculated to quantify its expression abundance and variations, using RSEM[6] software.

The FPKM formula is shown as follows:

$$FPKM = \frac{10^6 C}{NL/10^3}$$

Given FPKM(A) to be the expression of gene A, C to be number of fragments mapped to gene A, N to be total number of fragments that mapped to reference genes, and L to be number of bases on gene A. The FPKM method is able to eliminate the influence of different gene lengths and sequencing data amount on the calculation of gene expression. Therefore, the calculated gene expression can be directly used for comparing the difference of gene expression among samples.

2.5 Relationship analysis of samples

2.5.1 Correlation Analysis of Replicas

Correlation analysis was performed by R. Correlation of two parallel experiments provides the evaluation of the reliability of experimental results as well as operational stability. The correlation coefficient between two replicas was calculated to evaluate repeatability between samples. The closer the correlation coefficient gets to 1, the better the repeatability between two parallel experiments.

2.5.2 Principal Component Analysis

Principal component analysis (PCA) was performed with R package gmodels (http://www.r-project.org/) in this experience. PCA is a statistical procedure that converts hundreds of thousands of correlated variables (gene expression) into a set of values of linearly uncorrelated variables called principal components. PCA is largely used to reveal the structure/relationship of the samples/datas.

2.6 Differentially expressed genes (DEGs)

RNAs differential expression analysis was performed by DESeq2[7] software between two different groups (and by edgeR[8] between two samples). The genes/transcripts with the parameter of false discovery rate (FDR) below 0.05 and absolute fold change≥2 were considered differentially expressed genes/transcripts.

2.6.1 GO Enrichment Analysis

Gene Ontology (GO)[9] is an international standardized gene functional classification system which offers a dynamic-updated controlled vocabulary and a strictly defined concept to comprehensively describe properties of genes and their products in any organism. GO has three ontologies: molecular function, cellular component and biological process. The basic unit of GO is GO-term. Each GO-term belongs to a type of ontology.

GO enrichment analysis provides all GO terms that significantly enriched in DEGs comparing to the genome background, and filter the DEGs that correspond to biological functions. Firstly all DEGs were mapped to GO terms in the Gene Ontology database (http://www.geneontology.org/), gene numbers were calculated for every term, significantly enriched GO terms in DEGs comparing to the genome background were defined by hypergeometric test. The calculating formula of P-value is:

$$P = 1 - \sum_{i=0}^{m-1} \frac{\binom{M}{i}\binom{N-M}{n-i}}{\binom{N}{n}}$$

Here N is the number of all genes with GO annotation; n is the number of DEGs in N; M is the number of all genes that are annotated to the certain GO terms; m is the number of DEGs in M. The calculated p-value were gone through FDR Correction, taking FDR ≤ 0.05 as a threshold. GO terms meeting this condition were defined as significantly enriched GO terms

in DEGs. This analysis was able to recognize the main biological functions that DEGs exercise.

2.6.2 Pathway Enrichment Analysis

Genes usually interact with each other to play roles in certain biological functions. Pathway-based analysis helps to further understand genes biological functions. KEGG[10] is the major public pathway-related database. Pathway enrichment analysis identified significantly enriched metabolic pathways or signal transduction pathways in DEGs comparing with the whole genome background. The calculating formula is the same as that in GO analysis.

$$P = 1 - \sum_{i=0}^{m-1} \frac{\binom{M}{i}\binom{N-M}{n-i}}{\binom{N}{n}}$$

Here N is the number of all genes that with KEGG annotation, n is the number of DEGs in N, M is the number of all genes annotated to specific pathways, and m is number of DEGs in M. The calculated p-value was gone through FDR Correction, taking FDR ≤ 0.05 as a threshold. Pathways meeting this condition were defined as significantly enriched pathways in DEGs

2.6.3 Disease Ontology Enrichment Analysis (**only apply in humangenome reference**)

The Disease Ontology (DO)[11] has been developed as a standardized ontology for human disease with the purpose of providing the biomedical community with consistent, reusable and sustainable descriptions of human disease terms, phenotype characteristics and related medical vocabulary disease concepts. DO enrichment analysis identified significantly enriched human disease DO terms in DEGs comparing with the whole genome background. The calculating formula is the same as that in GO analysis.

$$P = 1 - \sum_{i=0}^{m-1} \frac{\binom{M}{i}\binom{N-M}{n-i}}{\binom{N}{n}}$$

Here N is the number of all genes that with DO annotation, n is the number of DEGs in

N, M is the number of all genes annotated to specific DO term, and m is number of DEGs in M. The calculated p-value was gone through FDR Correction, taking FDR ≤ 0.05 as a threshold. Do terms meeting this condition were defined as significantly enriched Do terms in DEGs

2.6.4 Reactome Enrichment Analysis (**only apply in human genome reference**)

The Reactome[12][13] is a free online database of biological pathways. The core unit of the Reactome data model is the reaction. Entities (nucleic acids, proteins, complexes and small molecules) participating in reactions form a network of biological interactions and are grouped into pathways. Examples of biological pathways in Reactome include signaling, innate and acquired immune function, transcriptional regulation, translation, apoptosis and classical intermediary metabolism.

Reactome enrichment analysis identified significantly enriched reactions in DEGs comparing with the whole genome background. The calculating formula is the same as that in GO analysis.

$$P = 1 - \sum_{i=0}^{m-1} \frac{\binom{M}{i}\binom{N-M}{n-i}}{\binom{N}{n}}$$

Here N is the number of all genes that with Reactome annotation, n is the number of DEGs in N, M is the number of all genes annotated to specific reactions, and m is number of DEGs in M. The calculated p-value was gone through FDR Correction, taking FDR ≤ 0.05 as a threshold. Reactions meeting this condition were defined as significantly enriched Reactions in DEGs

2.7 Gene Set Enrichment Analysis (GSEA)

We performed gene set enrichment analysis using software GSEA[14] and MSigDB[14] to identify whether a set of genes in specific GO terms\KEGG pathways\Reactome pathways \DO terms shows significant differences in two groups. Briefly, we input gene expression matrix and rank genes by SignaltoNoise normalization method. Enrichment scores and p

value was calculated in default parameters.

2.8 Protein-Protein interaction

Protein-Protein interaction network was identified using String v10[15], which determined genes as nodes and interaction as lines in a network. The network file was visualized using Cytoscape (v3.7.1)[16] software to present a core and hub gene biological interact.

2.9 Gene Structure Optimization **(not available in human reference)**

The gene structure and annotation for model organisms such as people, mice and Arabidopsis thaliana are almost complete, but for other species, the reads can be used for optimizing their gene structure, thus perfecting their gene annotations. After mapping reads to reference genome, the Hisat2 was used in reconstruction of transcripts which may extend the 5' untranslated region (5'UTR) or 3'UTR of gene to optimize the gene structure.

2.10 Single-nucleotide Polymorphism (SNP) Analysis

The GATK [17] (version 3.4-46) was used for calling variants of transcripts, and ANNOVAR was used for SNP/InDel annotation. The function, genome site and type of variation of SNPs were also analyzed.

2.11 RNA editing

RNA editing refers to variants on the mRNA level.

We use the following criteria to screen reliable editing sites from SNP sites [18] [19]:

1) Removing the low quality SNPs while calling SNP byGATK.
2) Correcting the SNPs around InDel region.
3) Choosing non-overlapping SNPs in UTR and EXON region.
4) Choosing SNPs with reference reads>=2 and variate reads>=3.

5) Choosing SNPs with the mutation frequency between 0.1 and0.9.

2.12 Alternative Splicing Analysis

The software rMATS [20](version 4.0.1) (http://rnaseq-mats.sourceforge.net/index.html) was used to identify alternative splicing events and analyze differential alternative splicing events between samples. We identified AS events with a false discovery rate (FDR) <0.05 in a comparison as significant AS events.

The classification of alternative splicing is as follows:

- SE: skipped exon
- MXE: mutually exclusive exon
- A5SS: alternative 5' splicesite
- A3SS: alternative 3' splicesite
- RI: retained intron

3 Reference

[1] Chen S, Zhou Y,Chen Y,et al. fastp: an ultra-fast all-in-one FASTQ preprocessor[J]. bioRxiv, 2018: 274100.
[2] Langmead B, Salzberg S L. Fast gapped-read alignment with Bowtie 2[J].Nature methods, 2012, 9(4): 357-359.
[3] Kim D, Langmead B, Salzberg S L. HISAT: a fast spliced aligner with low memory requirements[J]. Nature methods, 2015, 12(4): 357.
[4] Pertea M, Pertea G M, Antonescu C M, et al. StringTie enables improved reconstruction of a transcriptome from RNA-seq reads[J]. Nature biotechnology, 2015, 33(3): 290.
[5] Pertea M, Kim D, Pertea G M, et al. Transcript-level expression analysis of RNA-seq experiments with HISAT, StringTie and Ballgown[J]. Nature protocols, 2016, 11(9): 1650.
[6] Li B, Dewey CN. RSEM: accurate transcript quantification from RNA-Seq data with or without a reference genome. BMC Bioinformatics. 2011;12:323. Published 2011 Aug 4. doi:10.1186/1471-2105-12-323
[7] Love M I, Huber W, Anders S. Moderated estimation of fold change and dispersion for RNA-seq data with DESeq2[J]. Genome biology, 2014, 15(12): 550.
[8] Robinson M D, McCarthy D J, Smyth G K. edgeR: a Bioconductor package for differential expression analysis of digital gene expression data[J]. Bioinformatics, 2010, 26(1): 139-140.
[9] Ashburner M, Ball C A, Blake J A, et al. Gene ontology: tool for the unification of biology[J]. Nature genetics, 2000, 25(1): 25.
[10] Kanehisa M, Goto S. KEGG: kyoto encyclopedia of genes and genomes[J]. Nucleic acids research, 2000, 28(1): 27-30.
[11] Schriml L M, Arze C, Nadendla S, et al. Disease Ontology: a backbone for disease semantic integration[J]. Nucleic acids research, 2011, 40(D1): D940-D946.

[12] Croft D, O'kelly G, Wu G, et al. Reactome: a database of reactions, pathways and biological processes[J]. Nucleic acids research, 2010, 39(suppl_1): D691-D697.
[13] Fabregat A, Jupe S, Matthews L, et al. The reactome pathway knowledgebase[J]. Nucleic acids research, 2017, 46(D1): D649-D655.
[14] Subramanian A, Tamayo P, Mootha V K, et al. Gene set enrichment analysis: a knowledge-based approach for interpreting genome-wide expression profiles[J]. Proceedings of the National Academy of Sciences, 2005, 102(43): 15545-15550.
[15] Szklarczyk D, Franceschini A, Wyder S, et al. STRING v10: protein–protein interaction networks, integrated over the tree of life[J]. Nucleic acids research, 2014, 43(D1): D447-D452.
[16] Shannon P, Markiel A, Ozier O, et al. Cytoscape: a software environment for integrated models of biomolecular interaction networks[J]. Genome research, 2003, 13(11):2498-2504.
[17] Van der Auwera G A, Carneiro M O, Hartl C, et al. From FastQ data to high - confidence variant calls: the genome analysis toolkit best practices pipeline[J]. Current protocols in bioinformatics, 2013, 43(1): 11.10. 1-11.10. 33.
[18] Gokul R., Rui Z., Robert P.Identifying RNA editing sites using RNA sequencing data alone, Nat Methods. 2013 February; 10(2): 128–132.
[19] Jae Hoon B., Jae-Hyung L., Gang Li, Accurate identification of A-to-I RNA editing in human by transcriptome sequencing, Genome Res. 2012 22: 142-150.
[20] Shen S, Park J W, Lu Z, et al. rMATS: robust and flexible detection of differential alternative splicing from replicate RNA-Seq data[J]. Proceedings of the National Academy ofSciences, 2014, 111(51): E5593-E5601.
