# Supplementary figures and images for: Important nutrient sources and carbohydrate metabolism patterns in the growth and development of spargana
Source: Parasit Vectors. 2024 Feb 16;17:68. doi: 10.1186/s13071-024-06148-1 (PMC10873960; doi:10.1186/s13071-024-06148-1)

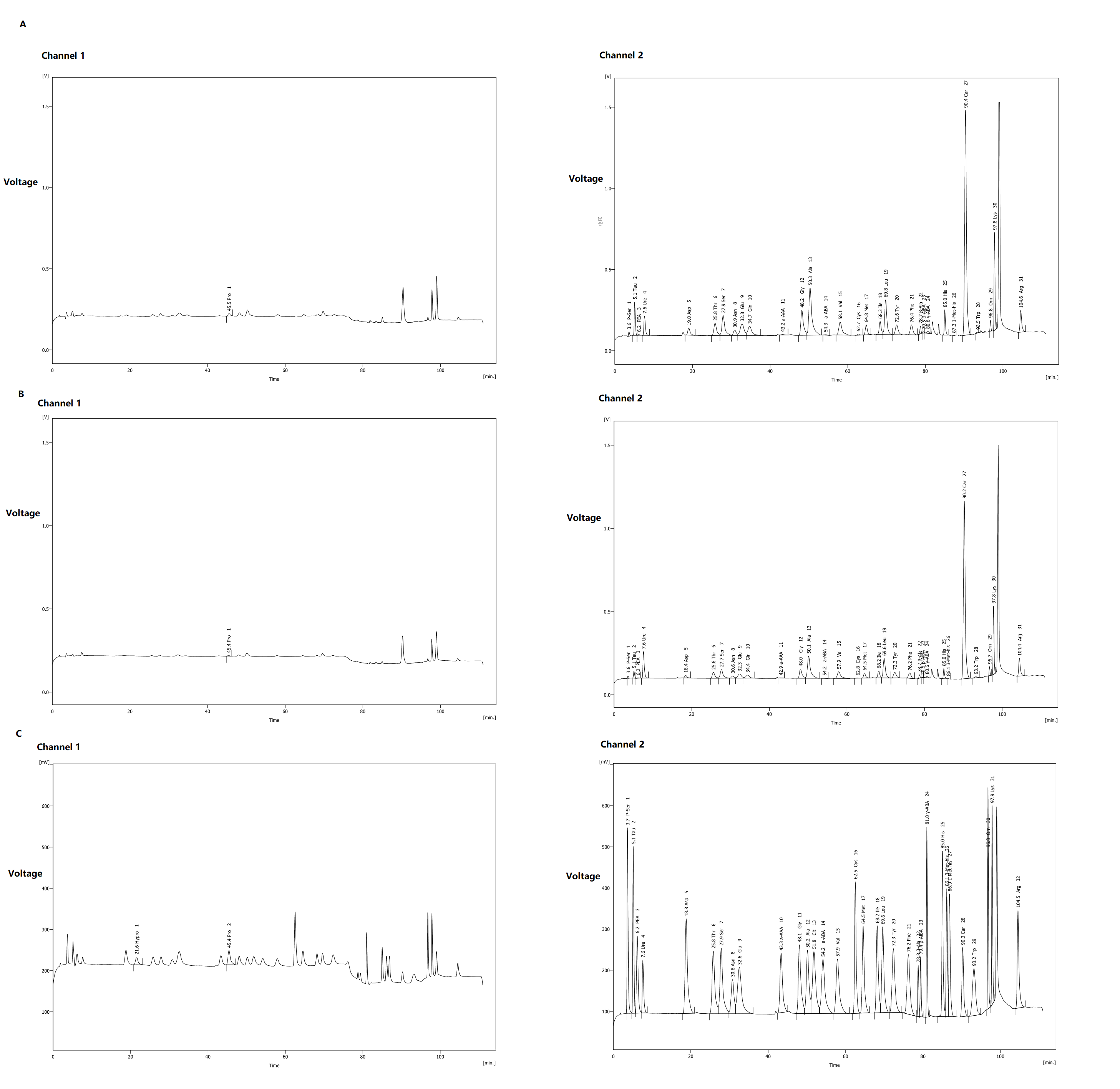

Supplement: Supplementary file 1 — Additional file 1: S1. Equations for calculation of the concentration of free amino acids in each. S2. The procedure of the transcriptome analysis applied in the study. S3. HPLC analytic profiles of amino acids in Fejervarya limnocharis. S4. HPLC analytic profiles of amino acids in Pelophylax plancyi. S5. STRING interaction diagram. PYGB: Myophosphorylase; ADCY9: Adenylate cyclase 9; GGT1: γ-glutamyltransferase 1; Amd2: S-adenosylmethionine decarboxylase; HDC :Histidine decarboxylase; GXYLT1: Glucoside xylosyltransferase 11; LAP2: Leucine aminopeptidase 2; AK2: Adenylate kinase 2; Ldhb: Lactate dehydrogenase B; GLUD1: Glutamate dehydrogenase 1. [file 13071_2024_6148_MOESM1_ESM.zip › Supplementary material/S 3. Fig Amino acid detection of R. limnocharis profiles.tif]

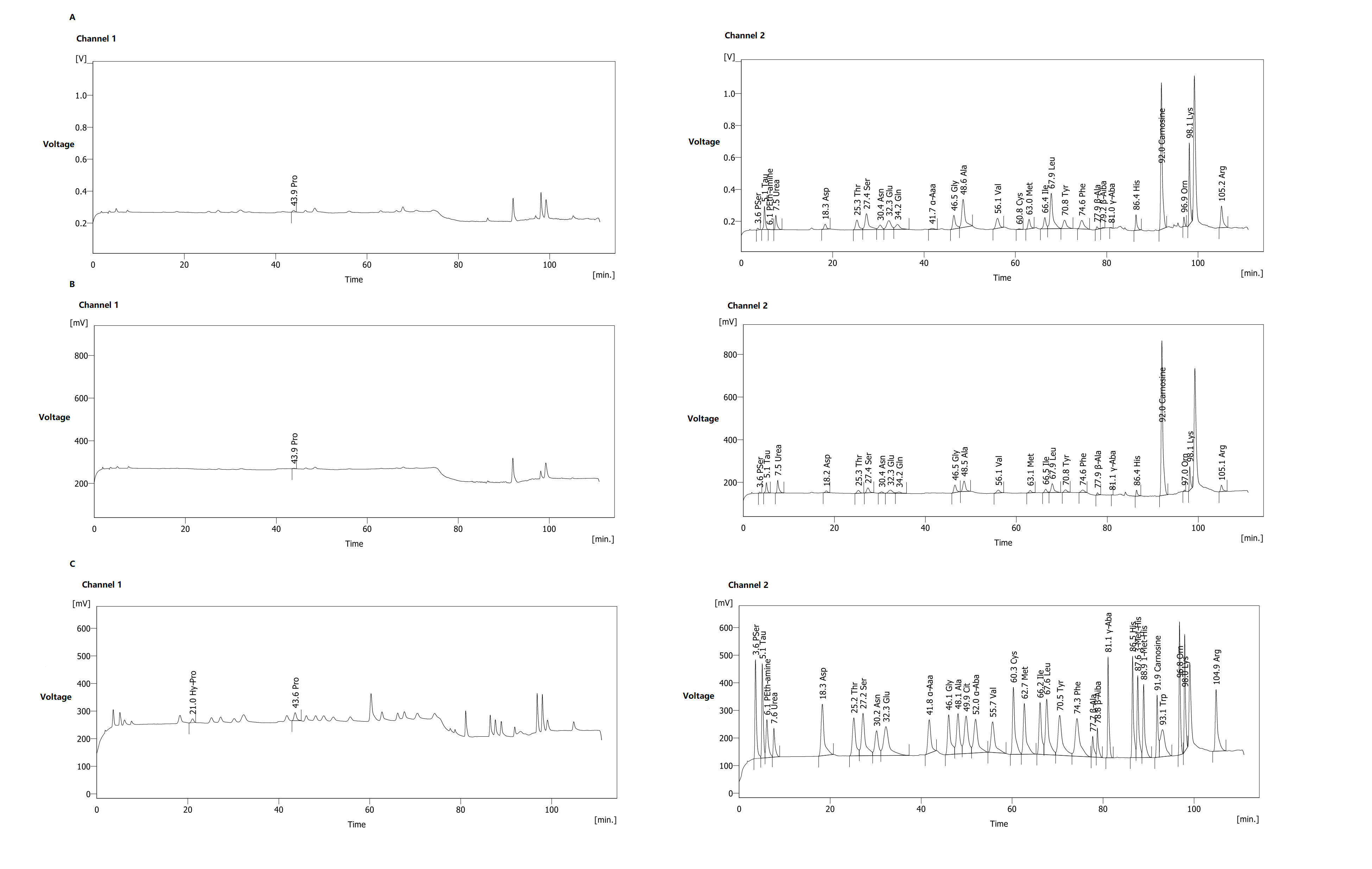

Supplement: Supplementary file 1 — Additional file 1: S1. Equations for calculation of the concentration of free amino acids in each. S2. The procedure of the transcriptome analysis applied in the study. S3. HPLC analytic profiles of amino acids in Fejervarya limnocharis. S4. HPLC analytic profiles of amino acids in Pelophylax plancyi. S5. STRING interaction diagram. PYGB: Myophosphorylase; ADCY9: Adenylate cyclase 9; GGT1: γ-glutamyltransferase 1; Amd2: S-adenosylmethionine decarboxylase; HDC :Histidine decarboxylase; GXYLT1: Glucoside xylosyltransferase 11; LAP2: Leucine aminopeptidase 2; AK2: Adenylate kinase 2; Ldhb: Lactate dehydrogenase B; GLUD1: Glutamate dehydrogenase 1. [file 13071_2024_6148_MOESM1_ESM.zip › Supplementary material/S 4. Fig Amino acid detection of R. plancyi profiles.tif]

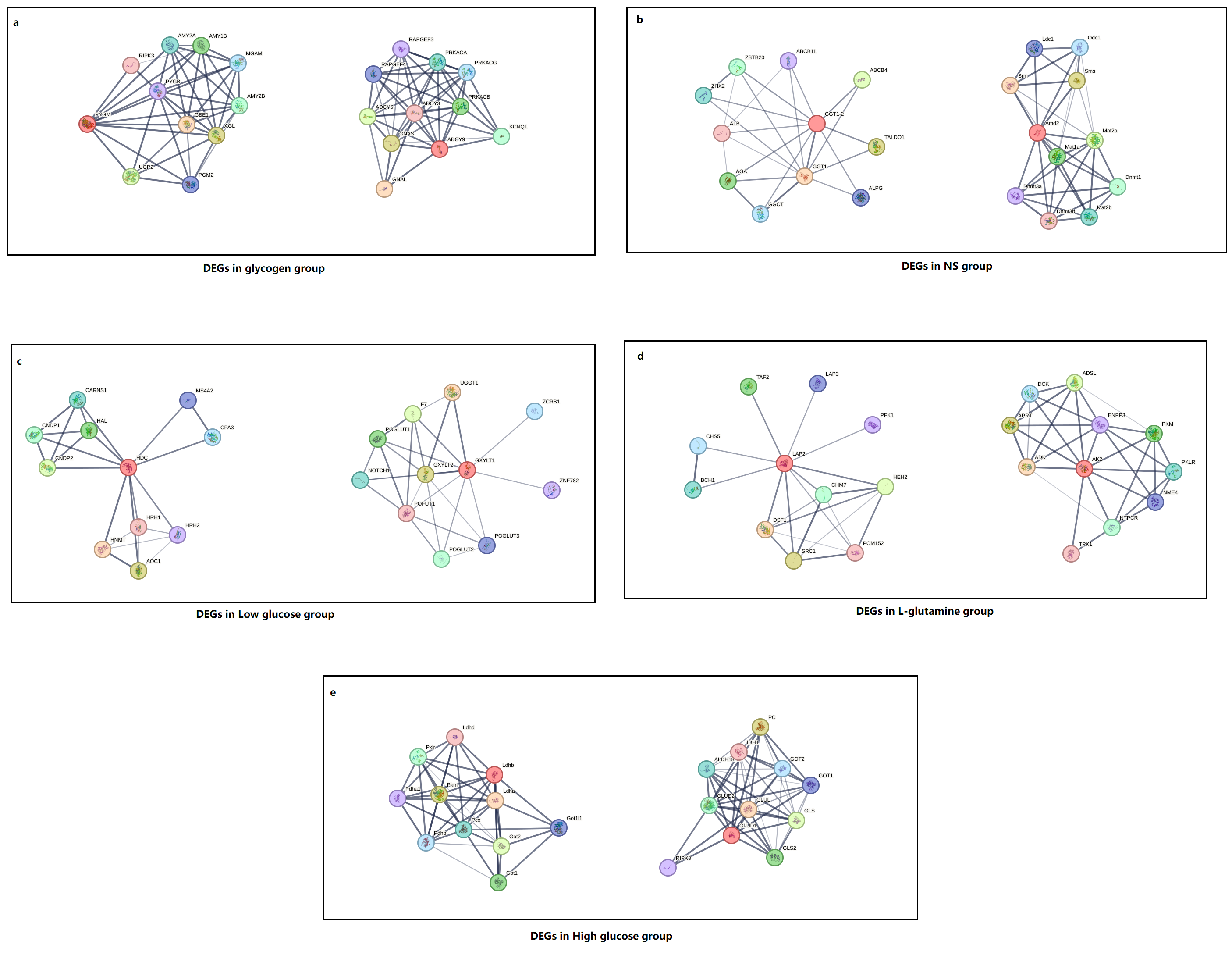

Supplement: Supplementary file 1 — Additional file 1: S1. Equations for calculation of the concentration of free amino acids in each. S2. The procedure of the transcriptome analysis applied in the study. S3. HPLC analytic profiles of amino acids in Fejervarya limnocharis. S4. HPLC analytic profiles of amino acids in Pelophylax plancyi. S5. STRING interaction diagram. PYGB: Myophosphorylase; ADCY9: Adenylate cyclase 9; GGT1: γ-glutamyltransferase 1; Amd2: S-adenosylmethionine decarboxylase; HDC :Histidine decarboxylase; GXYLT1: Glucoside xylosyltransferase 11; LAP2: Leucine aminopeptidase 2; AK2: Adenylate kinase 2; Ldhb: Lactate dehydrogenase B; GLUD1: Glutamate dehydrogenase 1. [file 13071_2024_6148_MOESM1_ESM.zip › Supplementary material/S 5. Fig The interaction diagrams were obtained from STRING analysis.tif]
